# Unveiling Stability Criteria of DNA-Carbon Nanotubes Constructs by Scanning Tunneling Microscopy and Computational Modeling

**DOI:** 10.1155/2011/415621

**Published:** 2011-03-20

**Authors:** Svetlana Kilina, Dzmitry A. Yarotski, A. Alec Talin, Sergei Tretiak, Antoinette J. Taylor, Alexander V. Balatsky

**Affiliations:** ^1^Department of Chemistry and Biochemistry, North Dakota State University, Fargo, ND 58108-6050, USA; ^2^Los Alamos National Laboratory, Center for Integrated Nanotechnologies, Los Alamos, NM 87545, USA; ^3^NIST Center for Nanoscale Science and Technology, Energy Research Group, Gaithersburg, MD 20899, USA; ^4^Los Alamos National Laboratory, Theoretical Division, Los Alamos, NM 87545, USA

## Abstract

We present a combined approach that relies on computational simulations and scanning tunneling microscopy (STM) measurements to reveal morphological properties and stability criteria of carbon nanotube-DNA (CNT-DNA) constructs. Application of STM allows direct observation of very stable CNT-DNA hybrid structures with the well-defined DNA wrapping angle of 63.4° and a coiling period of 3.3 nm. Using force field simulations, we determine how the DNA-CNT binding energy depends on the sequence and binding geometry of a single strand DNA. This dependence allows us to quantitatively characterize the stability of a hybrid structure with an optimal *π*-stacking between DNA nucleotides and the tube surface and better interpret STM data. Our simulations clearly demonstrate the existence of a very stable DNA binding geometry for (6,5) CNT as evidenced by the presence of a well-defined minimum in the binding energy as a function of an angle between DNA strand and the nanotube chiral vector. This novel approach demonstrates the feasibility of CNT-DNA geometry studies with subnanometer resolution and paves the way towards complete characterization of the structural and electronic properties of drug-delivering systems based on DNA-CNT hybrids as a function of DNA sequence and a nanotube chirality.

## 1. Introduction

The development of highly specific drug delivery systems (DDSs) holds a great promise for increased therapeutic treatment efficiency and elimination of often harmful side effects. However, it is a formidable task due to additional strict requirements posed on DDS, such as high stability, ability to penetrate cellular membranes, and low cytotoxicity. Several important breakthroughs have been achieved in recent years using biologically inspired liposome, ligand, and antibody-based DDS, some of which are already used in clinical environment for cancer treatment with positive results [1, 2]. Despite initial success, these results provide only a hint of the potential capabilities of properly designed drug carriers, and further improvements of DDS are necessary, including yet another leap in specificity and better drug-load capacity. 

Recently, inorganic nanomaterials, such as carbon nanotubes (CNTs), nanowires, and metal or semiconductor nanoparticles, have attracted much attention due to their remarkable physical and chemical properties and, especially, the tunability of these properties provided by the system size. Unique functionality makes these nanoscale entities very attractive for applications in a wide range of biological and chemical problems, and, specifically, in the development of drug carrying platforms [3]. So far, the majority of preclinical studies of nanomaterial-based DDS have focused on oncology, thus making cancer the primary candidate for future clinical trials of these DDS. For example, gold nanoparticles have been extensively used to selectively precipitate in cancer cells and subsequently destroy them through laser light absorption and generation of large intracellular heat loads [4].

Among all the novel DDSs, however, CNTs appear to be one of the most promising materials. This view is rationalized by many potential advantages of functionalized CNTs over other types of DDS developed for cancer therapy [5]. First of all, CNTs feature high surface-to-volume and length-to-diameter ratios, allowing large drug loads while still being small enough to penetrate cellular walls. Second, CNT functionalization with various binding agents provides virtually unlimited tunability of binding specificity. Several research groups have already demonstrated that CNTs coated with lipid conjugates [6], copolymers, and surfactants [7] can deliver various molecular loads through cellular membranes *in vivo* and *in vitro* with high targeting specificity and low cytotoxicity [8, 9]. Third, the unique optical properties of CNTs permit efficient electromagnetic stimulation and highly sensitive detection of CNT-based DDS using various imaging modalities. For example, strong light absorption in the cell transparency region (0.7–1.1 *μ*m) allows CNTs to serve as a local heat source inside a target cell [10] or to be remotely triggered to release some of its drug-load with high spatial, temporal, and chemical selectivity [11, 12]. Driven by the intense global research to take advantage of the unique properties of CNTs, the use of CNTs in medicine has started to shift from proof-of-principle experiments to preclinical trials in a variety of therapeutic applications. Nevertheless, we still need to develop a better understanding of CNT functionalities in order to fully exploit all the potential benefits of CNTs in drug delivery and diagnostics and to assess the risks and benefits of these DDS.

One of the prominent ways to improve delivery specificity, DDS stability, and cell penetration reliability is functionalization of the nanotube surface with single-stranded DNA. Such CNT-DNA hybrids are widely used for biological sensing [13–15], as well as for separating CNTs based on dimensions and conductivity [16, 17]. CNT-DNA hybrids promise significant advantages over existing DDS: (i) adsorbed DNA strands remarkably improve the dispersibility of the nanotubes in water and biologically compatible buffers, while simultaneously offering a stable and flexible platform for further derivatization and binding agent attachment. (ii) The DNA strands also provide very stable interaction with CNT surface and help to control the length of the tubes [18]. Because DNA-CNT binding energy is fairly large, “fortification” of the nanotube segments covered by DNA is expected. When the CNT breakage occurs (e.g., because of thorough sonication [17]), it will take place in the regions with a lower tensile strength, that is, the uncovered parts of the nanotube, leaving the tubes of the length of the DNA-wrapped structures. All these features are very important for medical applications, since it has been shown that shortened, better isolated and dispersed, functionalized CNTs demonstrate an improved toxicological profile in *in vivo* studies [19–22].

It is widely recognized that structural and surface characteristics of DDS should critically influence their biological performance. Yet little is known about the detailed structure of CNT-DNA hybrids. Different computational approaches reported in the literature predict a large variation of the possible DNA binding geometries [23] from linear DNA alignment along the CNT [24] to wrapping of DNA around the CNT [25], with a finite probability of the DNA insertion into the interior volume of the CNT [26, 27]. In addition, recent experimental studies have empirically demonstrated that DNA oligomers with a particular sequence prefer to form stable structures with a specific kind of nanotubes and ignore others. These observations suggest that the chemical structure of DNA and the chirality of the CNT play a significant, if not determining, role in establishing the final hybrid geometry [16, 28, 29]. Unfortunately, the current theoretical framework cannot explain the wide geometry variations and sequence selectivity of the DNA-CNT binding. Additional complexity comes from the lack of understanding of the exact mechanisms of cellular membrane penetration by CNTs [12, 30]: it is unclear how the given hybrid structure influences penetration efficiency, as well as how the penetration process influences stability of a hybrid DDS. 

As such, new methods have to be developed for reliable prediction of the properties of DDS based on CNT-DNA hybrids and accurate control of drug binding and delivery. Considering its importance, the stability of DNA coating of the nanotube surface has to be analyzed in order to avoid the risk of macromolecule desorption or exchange with serum proteins and other blood components following administration. Hence, theoretical modeling and simulations capable of describing the DNA-CNT binding mechanisms and predicting the hybrid stable structure and its relevant properties will significantly benefit experimental *in vitro* and *in vivo* studies of CNT-DNA-based DDS. Such studies will also require an application of high-resolution nanoscale probes to test the theoretical predictions, visualize the DDS geometries, and provide feedback for gradual modeling refinement.

Here, we report on such a combined approach that involves, first, modeling to determine the stability criteria for CNT-DNA hybrid binding and, second, scanning tunneling microscopy (STM) for simultaneous structural and electronic characterization of hybrid structure and electronic properties with subnanometer resolution. We present the observed topographic images of the CNT-DNA hybrids with highly resolved morphological details. The STM images reveal very stable hybrid structures where DNA is wrapped around the CNT with a well-defined wrapping angle of 63.4° and a coiling period of 3.3 nm. Our previous studies [18, 31] of the helical nature of the charge density distribution in the nanotubes have demonstrated a strong correlation between CNT chirality and DNA wrapping geometry. In the current work, we further investigate this correlation and describe the dependence of the DNA-CNT binding energy on the chemical structure and wrapping geometry of a single strand DNA (ssDNA) around the (6,5) CNT. This information allows quantitative characterization of the stability of the hybrid structure with an optimal *π*-stacking between ssDNA bases and the nanotube surface. Our simulations clearly show the existence of a very stable DNA binding geometry for the (6,5) CNT which is determined by a strong dependence of the binding energy on angular detuning of DNA strand from the nanotube chiral vector. Finally, we provide the additional evidence that the stable binding geometry of DNA nucleotides and CNTs arises from the *π*-stacking interactions, which tend to align the molecular plane of nucleotide parallel to the tube surface.

## 2. Experimental Details

We used surfactant-based nanotube suspensions that were prepared by 2.5 hours of sonication of purified single-walled CNT (SWCNT) powder obtained from SES Research in 1% by weight of Triton X-100 in water. The final concentration of SWCNTs was ~0.1 mg/ml. To form DNA-based nanotube suspensions, a 20-mer DNA sequence of 5′NH_2_(C-6) GAGAAGAGAGCAGAAGGAGA-3′ was diluted to approximately 5 mg/ml in phosphate buffer solution with pH 7.4 (PBS 7.4). One mg of SWCNT was dissolved in approximately 250 microliters of the DNA solution and then diluted to approximately 0.75 ml with PBS 7.4. The resulting mixture was sonicated at 0°C for at least 90 min and then centrifuged at 14000 rpm for 90 min. 0.5 ml of the DNA/SWCNT solution was decanted and purified over a NAP-10 column using deionized water as the buffer, with only first 1/2 of the eluted volume being collected. The filtered solution was finally passed again through the NAP-10 column with deionized water as eluent.

**Figure 1:**
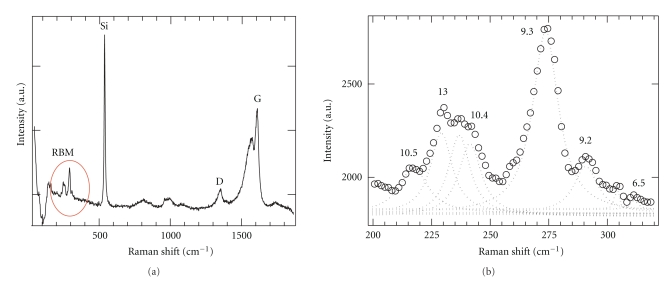
Raman spectra of the prepared DNA-CNT solution. (a) The wide frequency window showing all vibronic bands. (b) The frequency range associated with RBM bands of nanotubes.

As shown in Figure 1, Raman spectra of thus prepared solution contain six major radial breathing mode (RBM) frequencies, which can be attributed to (13,0), (10,4), (9,3), (9,2), (6,5), and (10,5) tubes [32]. The (9,3) tubes have the highest RBM intensity and, therefore, seem to be the most common type in the sample. It is known that functionalization of the tubes with DNA increases the optical response of CNTs due to enhanced dispersion and isolation of DNA-coated tubes [28, 29]. However, the high intensity of the Raman peak associated with (9,3) species is not necessary the sign of the preferable DNA attachment to the (9,3) tubes and most likely originates from the higher concentration of these tubes in the original solution.

**Figure 2:**
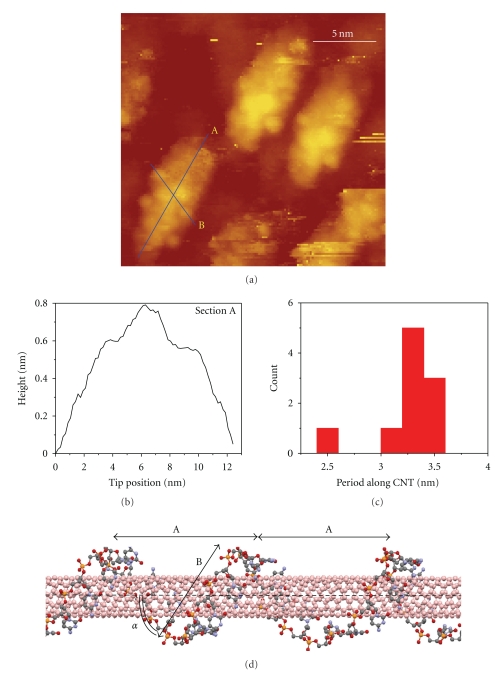
STM data and theoretical interpretation: (a) 21 × 21 nm STM topographic image of CNT-DNA hybrids on Si(110) substrate acquired at *I*_*t*_ = 10 pA and *U*_*b*_ = 3 V at 50 K; (b) height profile along Section A; (c) statistical distribution of characteristic lengths of periodic modulations extracted from height profiles along the Section A. (d) Optimized structures of (6,5) tube wrapped in GAGAAGAGAGCAGAAGGAGA-oligomer. For the simulated geometry, the average period of DNA helices along the tube is  *A* = 3.0–3.3 nm and the wrapping angle is *α* ~ 63°, which are in good agreement with an STM experiment.

After Raman characterization, a small drop of the CNT-DNA solution was deposited onto p-doped Si(110) substrate and allowed to dry. The samples were then transferred into the STM vacuum chamber and are annealed at 550°C for 30 min in order to remove the organic residue and the freshly formed oxide layer from the Si surface. Even though CNT-DNA hybrids in aqueous solution are unstable above 80°C, the critical temperature for the same constructs adsorbed onto Si(110) surface appears to be much higher, and heating up to 550°C does not destroy samples. Although the mechanism of such an improved thermal stability of CNT-DNA hybrids is not clear yet, we assume that a strong *π*–*π* interaction between the CNT surface and DNA bases is responsible for this stability, when it is not disturbed and screened by solvent interactions. A commercial UHV variable-temperature STM system (RHK Technology Inc., UHV300) was used to obtain the topographic images of CNT-DNA hybrids shown in Figure 2(a). All measurements were performed at a pressure of 2 × 10^−10^ Torr and a temperature of 50 K.

## 3. Theoretical Modeling and Computational Details

We have chosen a specific (6,5) nanotube for hybrid structure simulations since it provides the best match to the STM results, as was discussed in our previous studies of the CNT-DNA structures [18]. We use force field calculations to determine detailed geometrical features of an ssDNA adsorbed on the (6,5) SWNT (diameter of 0.8 nm and the chiral angle of 27°). Two configurations of the (6,5) SWNT are considered: with the length of three (~12 nm) and four (~16 nm) nanotube repeat units. To model the DNA adsorption on the CNT surface, we use an experimental 20-mer DNA sequence of 5′-GAGAAGAGAGCAGAAGGAGA-3′ and homogeneous ssDNA oligonucleotides with 23, 25, 29, 31, and 42 cytosine bases (C-23-mer, C-25-mer, C-29-mer, and C-31-mer and C-42-mer, resp.) and 25 guanine bases (G-25-mer). The size of the ssDNA is chosen to be shorter than the tube length to avoid interactions of the DNA with the tube edges.

**Figure 3:**
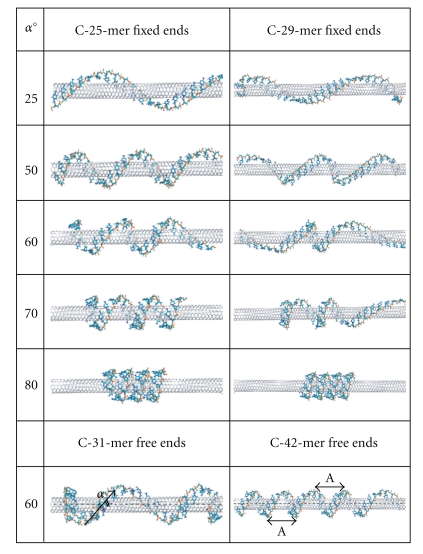
Optimized geometries of the (6,5) tube with adsorbed C-mers obtained from different initial wrapping configurations. First column shows the averaged final wrapping angle *α* of the DNA. Second and third columns correspond to hybrid configurations constructed from the 3 and 4 repeat units long (6,5) nanotube and DNA consisting of 25 (C-25-mer) and 29 (C-29-mer) cytosine bases, respectively. The bottom panel shows 31 and 42 C-mers wrapped along (6,5) tube of 4 units in length.

To construct different CNT-DNA hybrid configurations, the ssDNA is wrapped around the tube at angles varying from 10° to 80° with respect to the tube axis, as illustrated in Figure 3. For the initial configurations of the homogeneous ssDNA on the SWNT surface, we start with a single DNA unit consisting of a DNA base attached to a phosphate-deoxyribose molecule. First, we optimize the initial unit on the tube surface by placing it at a random angle *α* with respect to the tube axis. The coordinates of each atom *i* of the optimized unit are defined as (*x*_*i*_^*n*^, *y*_*i*_^*n*^, *z*_*i*_^*n*^), where the index *n* is the number of the unit (*n* = 0 for the initial unit). Subsequent DNA bases (*n* = 1,2, 3,…) are added as the replicas of the first adsorbed unit but are shifted along the tube axis by Δ*z* and twisted by the angle *ϕ*_*i*_^*n*^. Defining the size *L* of the unit as the distance between terminated atoms in the DNA base, the single increment along *z* is Δ*z* = *L* sin *α*. Then, the *z*-coordinates of each DNA atom of the next unit *n* satisfy the equation *z*_*i*_^*n*^ = *z*_*i*_^0^ + *n*Δ*z*, while *x*_*i*_^*n*^ and *y*_*i*_^*n*^ can be obtained from the coordinates *x*_*i*_^0^ and *y*_*i*_^0^ of the corresponding DNA atoms from the initial unit by applying the rotational matrix

(1) $$\hat{V}\left(\phi_{i}^{n}\right)=\left[\begin{array}{cc} \cos\;\;\left(\phi_{i}^{n}\right) & -\sin\;\left(\phi_{i}^{n}\right)\\ \sin\;\left(\phi_{i}^{n}\right) & \cos\;\;\left(\phi_{i}^{n}\right) \end{array}\right].$$

Here, *ϕ*_*i*_^*n*^ = *z*_*i*_^*n*^/(*R* tan *α*) is the rotational angle of the *i*th base of the *n*th unit of the ssDNA. Thus, each atom of the DNA backbone is placed along the helix curve with a helical angle *α*, the DNA wrapping angle with respect to the tube axis. When *ϕ*_*i*_^*n*^ = 2*π*, the *z*-coordinate defines the period length of the DNA wrapping along the tube axis. *R* = *R*_0_ + Δ stands for the helix radius, where *R*_0_ is a tube radius and Δ ~ 0.33 nm is a typical distance between the tube surface and DNA molecules in the *π*-stacking geometry. As a next step, these initial configurations of (6,5) SWNT and ssDNA are further optimized to obtain energetically favorable morphologies. Compared to the initial geometries, the DNA wrapping angles undergo small changes during geometrical optimization. Thus, we obtain many conformations of CNT-DNA hybrids with various DNA wrapping angles.

It is known that potential energy surfaces of biomolecules are extremely complicated [33]. Therefore, there are many distinct local potential minima where the hybrid system can be trapped depending on its initial configuration during the optimization procedure. This suggests a strong dependence of the total energy of the system on the wrapping angle of the ssDNA around the tube. However, optimized configurations obtained by the method described above often have loops at the center or ends of the tube leading to a variation of a wrapping angle along the CNT, as shown in Figure 3 (right panel). To obtain a more homogeneous distribution of the DNA wrapping angles, we fix the very end bases of the DNA and let all other atoms of the DNA and the tube move freely during geometrical optimization. This allows us to compare the dependence of the binding energies on the wrapping angle for two cases—with free and fixed DNA ends.

The binding energy, that is, the strength of the interaction between the ssDNA and the tube, is calculated as the difference between the total energies of the optimized CNT-DNA hybrid, the optimized bare CNT, and the optimized isolated DNA molecule. To find the optimized geometry of an isolated ssDNA, the DNA configuration obtained from the optimization of the CNT-DNA hybrid geometry and subsequent removal of all the CNT atoms is used as an initial approximation for the force field energy optimization. Finally, the optimized DNA configuration with the smallest total energy is chosen as the final configuration of the isolated DNA molecule. All geometrical optimizations are performed by means of the HyperChem software package [34] using the CHARM27 force field approach [35, 36] and an energy convergence limit of 0.001 KCal/(Åmol).

## 4. Experimental Results

A characteristic STM image of the CNT-DNA sample is shown in Figure 2(a). The DNA-covered parts of the nanotube are visible as large island-like protrusions on a flat substrate surface. Three notable features of the samples are evident in Figure 2(a). First, all observed islands have similar structure. This suggests that either we are able to resolve the structure of only one type of CNT-DNA hybrids or else hybrids consisting of different SWNT types have the same geometry. However, the latter assumption contradicts previous experimental [16, 18, 28, 37] and theoretical [17, 25, 28, 38] results that demonstrated strong dependence of the DNA wrapping geometry on CNT chirality. Therefore, we conclude that only one type of CNT-DNA sample is observable due to the selectivity of the DNA wrapping with respect to the tube chirality.

Second, there are no uncovered ends of SWNTs visible in the image as one might expect from the length differences between a typical SWNT (~100's of nm) and 20-mer ssDNA. This discrepancy can be explained by the sonication step in the sample preparation procedure [18]. Previously, it was found that thorough sonication leads to multiple nanotube breakages resulting in significant nanotube length reduction [17]. In our case, DNA-covered segments serve as fortified islands along the nanotube length, causing the breaks to occur at the edges of such regions and leaving only short, 10–15 nm, fragments of the original SWNT for observation. This suggests that the length of the CNT-DNA hybrids can be controlled with some degree of precision by varying the length of the ssDNA-covered segments and subsequent thorough sonication. This observation might be important for medicinal application of these materials. For instance, there is good agreement between multiple preclinical studies that shortening of functionalized CNT helps to reduce cytotoxicity [5, 39]. 

Third, the STM image in Figure 2(a) and height profile in Figure 2(b) clearly demonstrate the coiling character of the DNA strand binding to the nanotube surface. Regular height modulations of the DNA-covered segments of the CNTs are also visible in the image. Two sections of the hybrid profile emphasize the periodic nature of these modulations both along the nanotube (Section A) and across it (Section B). We attribute the three height peaks in Section A, Figure 2(b), to the three DNA coils lying on top of the nanotube surface. Indeed, the modulation depth of ~2 Å matches quite well an expected ~3 Å distance between the nanotube surface and the nucleotides that are aligned parallel to it in the *π*-stacking geometry [23, 25]. Section B represents the CNT-DNA hybrid profile variations in the direction of DNA coiling. Importantly, this section is oriented at a 63.4° angle with respect to the nanotube axis obtained in the same way as explained in [18]. This angle represents the DNA wrapping angle and should depend on the particular DNA sequence and the nanotube type, because nucleotides tend to arrange themselves on the nanotube surface in such a way as to minimize tension in the combined CNT-DNA system [33].

The overall observed width of the CNT-DNA composite is on the order of 5 nm. This value deviates significantly from the expected 2 ÷ 3 nm combined width of the CNT-DNA hybrid. The width of 2 ÷ 3 nm is expected due to the contribution of the CNT diameter of ~1-2 nm and DNA-CNT separation of ~0.3 nm (a typical *π*-stacking distance) on both sides of the CNT, as was discussed previously in [18]. We believe that DNA detachment from the nanotube sidewalls during annealing causes this discrepancy, increasing the overall hybrid width. The periodicity of the height profile in Section B also suggests that there are longitudinal DNA strand distortions that cannot be associated with any predicted binding stoichiometries [18]. However, it is impossible to directly detect the DNA detachment from the CNT surface using STM. The exposed CNT regions, if any occur during annealing, will protrude by about a nanometer and will not be accessible for direct imaging due to the cone-like shape of the STM tip.

To extract more quantitative information about the observed DNA wrapping geometry, we use the following procedure. First, cross-sections along the longitudinal axis of several SWNTs analogous to Section A in Figure 2(a) are taken. In this way, peaks in the topography can be attributed to the DNA strand, and dips represent the underlying SWNT surface between them. The Fourier transformation (FT) of such a section with respect to the longitudinal coordinate provides well-defined peaks in the spatial frequency domain due to the periodic nature of the profile variation, as shown in Figure 2(b). The characteristic length of the topographic height modulation is obtained by inversion of the spatial frequency of the corresponding peak maximum. Although observation of more cycles will provide higher accuracy in determining of the wrapping period, we believe that the precision achieved with three wrapping cycles observed in our experiments should suffice for comparison with the modeling results and nanotube identification. Indeed, experimental height modulation profile in Figure 2(b) can be approximated by the sine wave, and the width of the peak in the fast-FT spectrum of sine wave spanning *N* periods (*λ*) is ~2*λ*/*N* at zero level. Due to noise in the measured profiles, any point above 90% of the maximum peak amplitude level can be considered as a center peak frequency. However, it will result in only ~0.12*λ* spread of the measured period around the actual value, which in our case is ~0.3 nm. This error is much smaller than the difference between the wrapping periods for all the types of nanotubes present in the solution and should allow reliable separation of hybrids containing nanotubes of different chiralities as described below. The nanotube edges influence the DNA-CNT binding and, thus, the wrapping geometry. This causes small coil-to-coil distance variations, so that the DNA wrapping is not perfectly aligned with the nanotube chiral vector. However, these variations are on the order of 0.1-0.2 nm and fall well within the experimental error. Hence, they also can be neglected in the comparison of the modeled structure with the STM images.

Using this procedure, the dependence of the frequency of occurrence of a particular period on its magnitude for all hybrids in our images was extracted and is plotted in Figure 2(c). As can be seen, the characteristic period of the height variation along the CNT is 3.3 nm and represents the coiling period of the DNA strand around CNT. Thus, our STM images reveal the DNA wrapping angle of ~63° and the most probable DNA coiling period of ~3.3 nm.

## 5. Simulations Results and Discussion

Previous molecular simulations [33] predict that short ssDNA strands can adopt a number of helical conformations when placed on a nanotube. The geometries observed by STM here suggest an existence of very specific stable structure with the DNA helical period of 3.3 nm and the wrapping angle of ~63°. Our simulations of CNT-DNA hybrid constructed from the (6,5) tube and 20-mer ssDNA that was used in STM imaging have also resulted in a very stable configuration with the binding energy of −0.8 eV per base, wrapping angles of ~63°, and wrapping period of 3.0–3.3 nm, as shown in Figure 2(d). The optimized structure of the hybrid also confirms that the stable binding geometry of DNA nucleotides and CNT arises from the *π*-stacking interactions, which tend to align the nucleotide molecular plane parallel to the tube surface.

For further examination of the stability of different CNT-DNA hybrid structures, we calculated the binding energy between various adsorbed ssDNA C-mers and G-mers and the (6,5) tube at different wrapping geometries, as shown in Figure 3. It is obvious that the distribution of wrapping angles along the nanotube length is not homogeneous with most deviations occurring at the edges of the nanotube. For the fixed DNA geometries, when a few DNA bases at the ends are not free to move with other atoms of the systems during geometry optimization, the homogeneity of wrapping angles improves significantly; see Figure 3 (left panel). Overall, the deviation from a mean value of wrapping angle is about 10°–15° for the structures with fixed ends and up to 20°–30° for structures with free ends.

**Figure 4:**
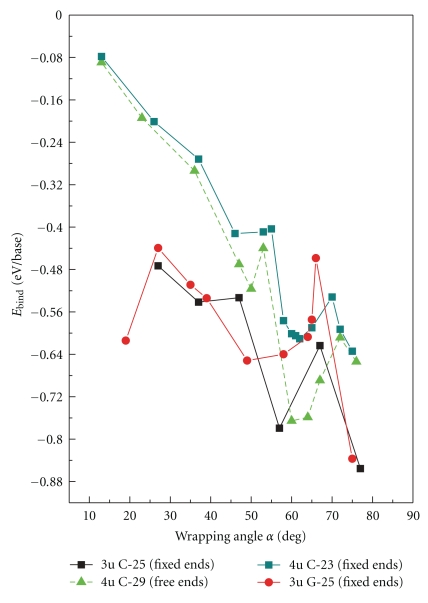
Variation of the binding energy of the CNT-DNA hybrids with the DNA wrapping angle. The solid lines correspond to hybrid configurations with fixed ends, that is, where the end bases of the DNA molecule are fixed and all other atoms of the hybrid system are free to move during geometry optimization. Dashed lines represent the optimized hybrid structure where all the atoms are allowed to move during optimization. The red line corresponds to the hybrid constructed out of 3 unit-long (6,5) tube (3u) and DNA strand consisting of 25 guanine bases (G-25); the black line represents the same tube but with 25-mer cythosine bases (C-25); the dark green line represents (6,5) tube of 4 repeat units in length (4u) with adsorbed 23-mer cythosine bases (C-23). The light green dashed line corresponds to configurations constructed from the (6,5) nanotube of 4 repeat units in length (4u) and 29-mer cythosine bases (C-29).

Figure 4 shows the binding energy of the DNA and the (6,5) SWNT as a function of the average wrapping angle. The minimum of the curve indicates the most stable hybrid configuration with the strongest interaction between the tube surface and the DNA strand. For all C-mers, a well-defined minimum is found in the range of 58°–63°; these wrapping angles correlate well with the chiral angle of the (6,5) tube. For the G-mer, the minimum is slightly shifted towards smaller angles of 50°–60°. For all hybrids we considered, the energy barrier around the minimum is about 0.2-0.3 eV, which is significantly higher than thermal fluctuation energies. The CNT-DNA interactions are also very substantial (−0.6 eV and −0.8 eV) implying very stable hybrid configurations for wrapping angles of 50°–63°. Thus, we conclude that hybrids with DNA wrapped in correlation with the (6,5) chirality of nanotube have extremely stable configurations. For these structures, ssDNA is unlikely to be detached from the tube because of external perturbations, such as ambient thermal vibrations, solvent effects, and exchanges with blood serum. All these observations point to the utility of DNA-functionalized CNT for medicinal purposes.

The smaller the wrapping angle of C-mers, the larger the energy, reflecting much weaker interaction of cytosine-oligomers with the CNT for these geometries. In contrast, G-mers provide very stable configurations not only at 50°–60° but also at small wrapping angles of 10°–20°. Interestingly, not all guanine molecules are oriented parallel to the tube surface at small wrapping angles, as observed for cytosine-oligomers: a few guanine bases have nearly normal orientation to the tube surface and form the *π*–*π* stacking with each other. This behavior most likely originates from a larger size of guanines compared to cytosines, which favors such interactions. The difference between C-mer and G-mer optimal wrapping angles, at which the most stable hybrid conformations occur, may explain a previously observed difference in stability of CNT-DNA hybrids with respect to the chemical structure/sequence of the adsorbed DNA.

For the large angles *α* > 70°, the binding energy decreases for both G-mer and C-mers. For the short tubes and short DNA oligomers, the binding energy at *α* ~ 75° becomes even smaller than that of configurations with ~60° angles. This decrease most likely originates from formation of additional bonds between DNA bases and the phosphate groups due to a very small separation of DNA loops on CNT surface; see Figure 3. Interestingly, such bonding is favored by the presence of the SWNT, since optimized configurations of an isolated DNA strand do not indicate similar tendency. If solvent media are introduced, formation of these hydrogen bonds will likely be suppressed by solvent-phosphate backbone interactions.

It is important to mention that structures with large wrapping angles result in much smaller wrapping periods of about 1 nm. The short wrapping periods, if present in the experimental samples, mean that the gaps between the DNA strands on the tube surface have to be also very small, on the order of 0.2–0.8 nm, as compared to ~2.2 nm observed in STM images. The fact that we have only observed geometries with ~63° wrapping angle in our experiments can be, thus, attributed to the inability of our instrument to resolve such small gaps. This is confirmed by the data presented in Figure 2(b), where dome-like modulation structure due to convolution of tip shape with sample structure is visible instead of expected 0.47 nm and 0.35 nm steps formed by the DNA backbone and nucleotides, correspondingly. 

## 6. Conclusions

Characterization of CNT-DNA hybrids using STM reveals a very stable structure of DNA binding to a single CNT where DNA wraps around the tube at 63° angle with a coiling period of 3.3 nm. To complement and help interpret STM measurements, we have performed force field simulations that provided insight into the energetic stability of CNT-DNA hybrids. The modeling results are in very good agreement with experimental observations and clearly show the existence of a stable DNA binding geometry to (6,5) SWNT as determined by the strong dependence of the binding energy on angular detuning of the DNA strand from the CNT chiral vector. The calculations also confirm that such a correlation between the DNA wrapping and nanotube chirality arises from optimization of *π*-stacking interactions between molecular orbitals of DNA bases and the *π* orbitals of the nanotube. Based on STM data and calculated stability criteria for different DNA conformations on the nanotube surface, we conclude that ssDNA wraps around the (6,5) tube in accordance to the tube chirality. Substantial binding energies of 0.6–0.8 eV and high energy barriers of 0.1–0.3 eV separating the hybrid configurations of coiled and uncoiled ssDNA imply an extreme stability of such hybrid systems. This result suggests that external disturbances caused by body heat, solvent effects, and exchanges with blood serum are highly unlikely to detach the DNA from the CNT surface. Therefore, CNT-DNA hybrids hold great promise for development of very reliable and stable DDS.

We also found that sonication of CNT-DNA hybrids leads to reduction of nanotube ends uncoated by DNA. Thus, we suggest that the length of the CNT-DNA hybrids can be reduced with a precise control by applying sonication and varying the DNA sequence length adsorbed on the tube surface. This observation might be important for medical application of these materials, since shortening of functionalized CNTs reduces their cytotoxicity.

Overall, our results demonstrate the feasibility of CNT-DNA geometry studies with subnanometer resolution and pave the way towards complete characterization of the hybrid structural and electronic properties as a function of DNA sequence and nanotube type. In addition, our combined approach can be used in the future to predict and characterize important properties of hybrid-based DDS and details of their interaction with the drug molecules, such as controlled drug release triggered by the heat or laser-induced unwrapping of DNA strand from the nanotube surface.
